# Effect of systemic lidocaine on postoperative quality of recovery, the gastrointestinal function, inflammatory cytokines of lumbar spinal stenosis surgery: a randomized trial

**DOI:** 10.1038/s41598-023-45022-5

**Published:** 2023-10-17

**Authors:** Yu Wu, Zhuoming Chen, Caimiao Yao, Houxin Sun, Hongxia Li, Xuyang Du, Jianzheng Cheng, Xiaojian Wan

**Affiliations:** 1 Department of Anesthesiology, Bethune International Peace Hospital, Shijiazhuang, 050082 China; 2 School of Textile and Fashion, Shanghai University of Engineering Science, Shanghai, 201620 China; 3 Department of Clinical Laboratory, Bethune International Peace Hospital, Shijiazhuang, 050082 China; 4 Department of Anesthesiology and Critical Care Medicine, Changhai Hospital, Naval Medical University, Shanghai, 200433 China

**Keywords:** Clinical trials, Randomized controlled trials

## Abstract

Surgery is one of the most frequent and effective intervention strategies for lumbar spinal stenosis, however, one-third of patients are not satisfied with postoperative outcomes. It is not clear whether perioperative systemic lidocaine could accelerate the early postoperative quality of recovery in patients undergoing lumbar spinal stenosis surgery. 66 patients were enrolled in this trial. Lidocaine or placebo was administered at a loading dose of 1.5 mg/kg for 10 min and then infused at 2.0 mg/kg/hour till the end of surgery. Continued infusion by postoperative patient-controlled intravenous analgesia with a dose of 40 mg/hour. The primary outcome was the quality of recovery. Secondary outcomes included the time of the patient's first flatus, catheter removal time, underground time from the end of the surgery, pain score, levels of inflammatory factors (IL-6, IL-10, TNF-α), postoperative nausea and vomiting (PONV), sufentanil rescues, patients’ satisfaction scores, and complications of lidocaine. Eventually, 56 patients were in the final analysis with similar age, Body Mass Index (BMI), duration of surgery and anesthesia, and median QoR-15 score (a development and Psychometric Evaluation of a Postoperative Quality of Recovery Score). The difference in median QoR-15 score in placebo versus lidocaine patients was statistically significant (IQR, 106 (104–108) versus 114 (108.25–119.25), *P* < 0.001). The Numeric Rating Scale (NRS) score at the 12th hour, median sufentanil rescue consumption, IL-6, tumor necrosis factor-alpha (TNF-α) of patients treatment with lidocaine were lower. Nevertheless, patients given lidocaine had high satisfaction scores. Suggesting that lidocaine enhanced the postoperative quality of recovery, met early postoperative gastrointestinal function recovery, provided superior pain relief, lessened inflammatory cytokines, etc., indicating it may be a useful intervention to aid recovery following lumbar spinal stenosis surgery.

## Introduction

Lumbar spinal stenosis (LSS) is a common chronic spinal disease, more experienced by people aged over 50 years^1^. A total of 266 million individuals (3.63% of the worldwide population) were found to have lumbar degenerative spine disease, there are more cases in high-income countries^2^. In the United States, more than half a million people over the age of 65 suffer from spinal stenosis^3^ and the fastest-growing age group is the 45 and older population^4^. The most common cause of spinal stenosis is natural aging and progressive degeneration of the spine^4^. Surgery is one of the most frequent and effective intervention strategies^5^. However, about one-third of patients with lumbar decompression are not satisfied with postoperative outcomes due to pain and inferior functional levels^6^.

Postoperative pain is one of the most common postoperative features of patients with lumbar spinal stenosis^7^. Postoperative inflammation is still regarded as an important criterion for evaluating the surgical outcome of patients with lumbar spinal stenosis. The ongoing chronic inflammation and subsequent fibrosis play an important role in patients with lumbar spinal canal stenosis^8^. Cytokines are small secreted proteins released by cells that specifically affect the interactions and communications between cells. Pro-inflammatory cytokines are produced predominantly by activated macrophages and are involved in the up-regulation of inflammatory reactions. There is abundant evidence that certain pro-inflammatory cytokines such as IL-1β, IL-6, and TNF-α are involved in the process of pathological pain^9^.

Lidocaine is one of the most widely used local anesthetics in clinics. Previous studies have shown that patients received a bolus of 1.5–2 mg/kg lidocaine completed within 10 min before the induction of anesthesia followed by continuous infusions of 2 mg/kg/hour lidocaine intravenously during anesthesia could improve QoR-40 scores in patients with abdomen surgery^10–12^, reduce the need for opioids and the intensity of postoperative pain, and extende the time to first request morphine^13^. Lidocaine affects inflammatory cells in vitro, such as by inhibiting priming of human peripheral poly-morphonuclear cells or neutrophils^14^. Lidocaine can furthermore reduce the release of mediators of inflammation, such as IL-4, IL-6, and TNF- α^15^. However, intraoperative infusions of lidocaine did not improve recovery in patients who had multilevel spine surgery^16^. However, it is not clear whether perioperative systemic lidocaine could accelerate the early phase of postoperative quality of recovery and the other effects in patients undergoing lumbar spinal stenosis surgery. We hypothesized that perioperative intravenous lidocaine infusion can significantly improve postoperative recovery quality and improve prognosis in patients with lumbar spinal stenosis surgery.

## Methods

### Study participants

The single-center, double-blind, placebo-controlled randomized clinical trial study enrolled patients who underwent lumbar spinal stenosis surgery from January 1, 2022, to June 30, 2022. This study was approved by the Ethics Committee of Bethune International Peace Hospital (No.2021-KY-165) and registered at the Chinese Clinical Trial Registry (ChiCTR2100054852, 28/12/2021). All participants gave written informed consent. All methods were carried out in accordance with relevant guidelines and regulations, following the Declaration of Helsinki. A total of 66 patients aged 18–75 years old with American Society of Anesthesiologists (ASA) grade I–III were scheduled for open posterior lumbar spinal fusion surgery under general anesthesia. The exclusion criteria included allergy to anesthetic drugs, coagulation abnormalities, preoperative gastrointestinal obstruction, severe cardiac disease, severe sinoatrial node dysfunction(degree II or III atrioventricular block), congestive heart failure, liver and kidney insufficiency or cognitive dysfunction, hypoxemia, hypoalbuminemia, a long history of anti-inflammatory drugs and hormones; The rejection criteria included patients who had serious complications or accidents during perioperative or anesthesia, not cooperating with the questionnaire and those who required admission to the Intensive Care Unit(ICU) after surgery.

### Randomization and masking

The study participants were randomly grouped on a scale of 1:1 using a computer-generated list of random numbers. The distribution results are sealed in an opaque envelope and kept by the study manager. On a surgical day, the study manager handed the envelope to the anesthesia assistant who dispensed the anesthetic fluid. The patients were randomly assigned to two groups: the saline placebo group and the lidocaine group, with 33 patients in each group.

Drugs were prepared in 20 ml syringes (for bolus administration) and another 20 ml syringes (for continuous infusion), containing 2% lidocaine solution or an equal amount of 0.9% normal saline. Postoperative patient-controlled intravenous analgesia (PCIA) with sufentanil and with or without lidocaine. The assistant handed a syringe full of liquid medicine or PCIA to the anesthesiologist without knowing which patient was in which group. Thus, group assignments are blinded by patients, healthcare providers, and data collectors.

### Standard procedure

Dynamic monitoring of vital signs, intravenous indent needles, non-invasive blood pressure, electrocardiogram (ECG), heart rate, pulse oxygen saturation, and bispectral index were monitored when the patient entered the operating room. The trial was designed with an initial lidocaine (Hunan Kelun Pharmaceutical Co., Ltd, F210625c) load of 1.5 mg/kg for 10 min, followed by an infusion rate of 2.0 mg/kg/hour until the end of the procedure^17^. The patient-controlled intravenous analgesia of 40 mg/hour was continuously injected postoperation for 48 hour. Patients in the placebo group received an equal-volume load dose of normal saline and a placebo infusion. The anesthesia induction regimen was as follows: midazolam (Jiangsu Nhwa Pharmaceutical Co., Ltd) 0.02 to 0.04 mg/kg, cis-atracurium(Hangzhou Hongyou Medical Technology Co., LTD) 0.2 mg/kg, sufentanil(Renfu Pharmaceutical Co., LTD) 0.2 to 0.3 μg/kg, propofol(Jiangsu Yingke biological pharmaceutical Co., LTD) 1.0 to 2.5 mg/kg intravenously. Endotracheal intubation after 120 seconds of muscle relaxants taking effect fully. The volume control ventilation was performed according to standard body weight (7 ~ 9 ml/kg). Maintain end-expiratory partial pressure of carbon dioxide (PetCO_2_) between 35 and 45 mmHg. General anesthesia was maintained by continuous intravenous injection of propofol at 50 to 100 μg/kg/min and remifentanil (Renfu Pharmaceutical Co., LTD) at 0.1 to 1.0 μg/kg/min in a mixture of sevoflurane(Shanghai Hengrui Pharmaceutical Co., LTD) oxygen/air at 2.0 to 3.0%. BIS was also maintained at 40 to 60^18^. Cis-atracurium 1 ~ 2 μg/kg/min in maintained neuromuscular block and (Train-of-four) TOF ratio (T4/T1) > 0.9. Both groups received an intravenous injection of 10 mg of tropisetron during the operation to prevent postoperative nausea and vomiting (PONV). Atropinewas given 0.5 mg when the patient's heart rate fell below 50 beats per minute, urapidil (Guangzhou Wanzheng Pharmaceutical Co., LTD) 5 mg when the patient's blood pressure rose above 20% of baseline, and deoxyadrenalin(Shanghai Hefeng Pharmaceutical Co., LTD) 50 μg when the patient's blood pressure fell above 20% of baseline. A mixture of sufentanil 5 μg, neostigmine 1 mg, and atropine 0.5 mg was administered intravenously at the end of epidermal sutures. The patient was then transferred to the post-anesthesia care unit (PACU) for care. The postoperative analgesia regimen was as follows: conventional intravenous parecoxib 40 mg, once every 12 hours, PCIA combined with sufentanil and lidocaine, pump rate constant, pump rate 2 ml/h, sufentanil 2 ug(2ug/ml) as needed, locked interval 15 min. Postoperative pain was scored on NRS ranging from 0 (no pain) to 10 (the most severe pain imaginable). If the NRS pain score exceeds 3 or the patient requests, 2 μg sufentanil intravenously administered with a PCIA device is used as a rescue analgesic. If PONV occurs, intravenous tropisetron 5 mg and metoclopramide 10 mg are administered as rescue antiemetics.

5 ml of blood was drawn from a peripheral vein before induction of anesthesia and 48 hours after surgery in all patients. Blood samples were centrifuged for 5 min at 10,000 rpm and stored in a refrigerator at − 80 °C for subsequent testing. Enzyme-linked immunospecific assay (ELISA) was used to test IL-6, IL-10, and TNF-α plasma concentrations by commercially available kits (eBioscience Co., Ltd., San Diego, USA). The incidence of patients with postoperative arrhythmia, dizziness, shivering, and adverse effects within 48 hours after surgery. The occurrence of neurogenic intestinal dysfunction (NID) and neurogenic bladder (NB) was recorded until the patient was discharged.

### Primary and secondary outcomes

This study was to determine whether perioperative systemic lidocaine is superior in improving the quality of rehabilitation after lumbar spinal stenosis surgery. The primary outcome was quality of recovery, measured 48 hours postoperatively using the Chinese version of QoR-15, a development and Psychometric Evaluation of a Postoperative Quality of Recovery Score, which comprises 5 domains of testing: pain, physical comfort, physical independence, psychological support, and emotional state^19^. The secondary outcomes included the time of the patient's first flatus, catheter removal time, underground time from the end of the surgery (both corrected to the nearest hour), pain score, and levels of inflammatory factors (IL-6, IL-10, TNF-α) before induction and 48 hours after the operation, postoperative salvage dosage of sufentanil, patients’ satisfaction scores and complications of lidocaine. The patients also were asked to report symptoms related to abnormal gastrointestinal function, including nausea and vomiting or abdominal bloating.

### Sample size estimation

The sample size was calculated based on global QoR-15 points 48 hours after surgery by StatBox: A Online Statistical Computing System. Biostatistics Team of CMT. URL https://www.biostats.cn/statbox/. A change of 8.0 on the QoR-15 score was considered a clinically significant improvement after surgery and anesthesia^20^. According to the pilot study, the QoR-15 scores at 48 hours postoperatively were equivalent to 116 (10) in the lidocaine group. Assuming a two-tailed alpha threshold of 0.05 and a power (1-beta) of 90% to detect an increment of 8.0 in the QoR-15 scores at a significance, 27 participants in each group were required. Taking into consideration a 20% withdrawal and loss for follow-up, we finally recruited 66 patients for this study.

### Statistical analysis

All statistical analyses were performed using the IBM SPSS software Version.23.0 (IBM, Armonk, NY: IBM Corp). The normality of quantitative variables was examined with the Kolmogorov–Smirnov test. Quantitative variables are expressed as mean or median (interquartile range, IQR). Student T-test was used to compare the mean values of age, weight, height, operation time, anesthesia time, PACU discharge time, cytokines, and so on. The overall QoR-15 score and the cumulative use of sufentanil after surgery are reported as the median (IQR) and were analyzed using the Manne-Whitney U-test. An estimated median difference and 95% CI of differences were given for each statistical comparison. Categorical variables were reported as numbers and percentages. The proportion of ASA classification and the number of PONV patients were compared by the χ2 test. Fisher's precise test was used to compare the groups’ dizziness, shivering, or headache rates^21^. In addition, an analysis of variance was performed on multiple comparisons to assess pain scores within 48 hours after surgery. A *P*-value less than 0.05 was considered statistically significant in the two-sided test.

## Results

**Figure 1:**
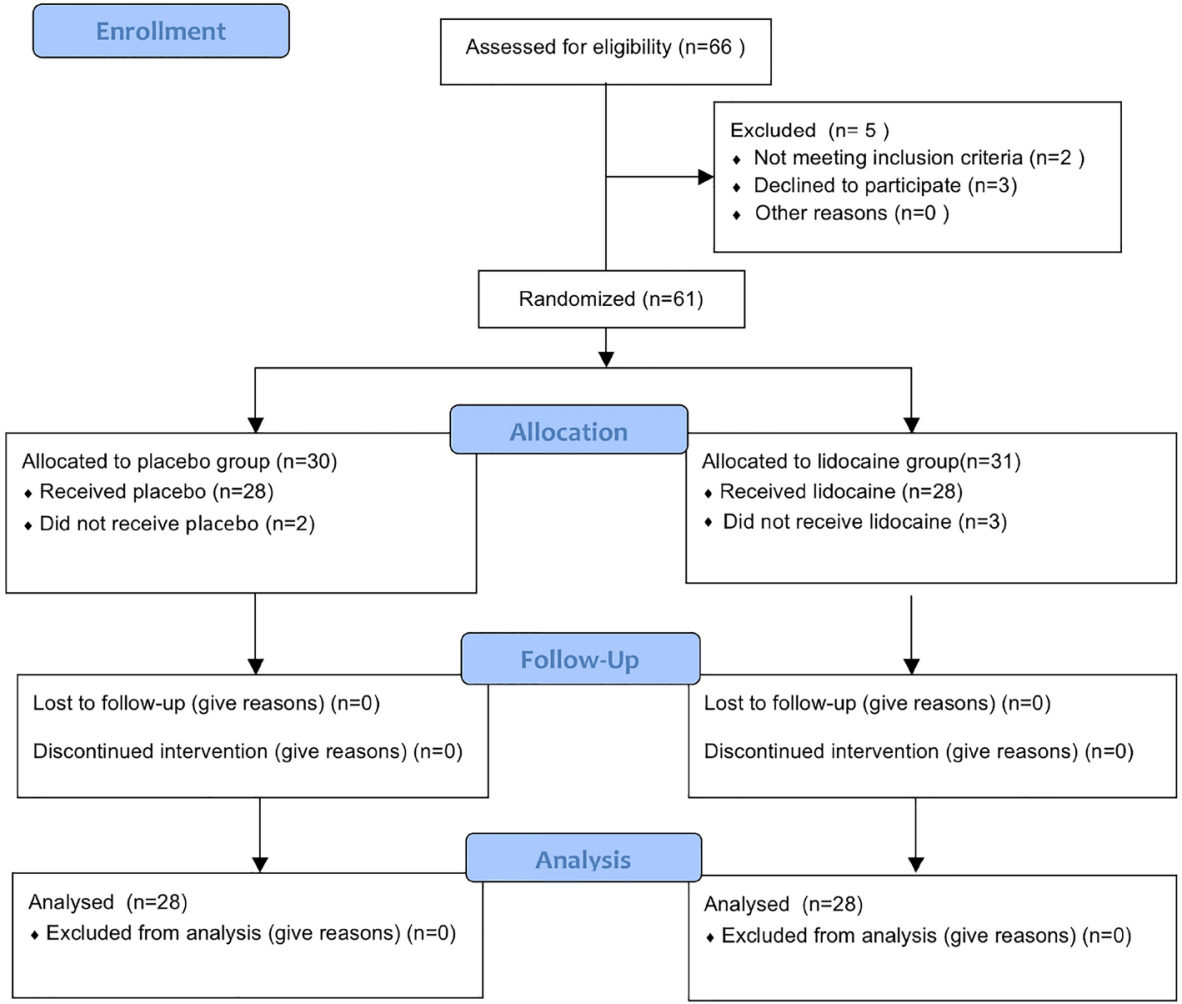
CONSORT 2010 Flow Diagram.

The CONSORT 2010 flowchart is shown in Fig. 1. Between January 1, 2022, to June 30, 2022, we screened 66 potential participants who planned an elective lumbar spinal stenosis surgery under general anesthesia. Based on inclusion criteria, 2 participants were deemed ineligible and 3 declined to participate. A total of 61 participants participated in the trial. After randomization, 2 patients in the placebo group and 3 in the lidocaine group were excluded for protocol violation. Therefore, data from 56 patients were used in the final analysis. Patient demographic characteristics were similar between the two groups (Table 1).

**Table 1:** Demographic characteristics.

Patient characteristics	Placebo(n = 28)	Lidocaine (n = 28)	t or χ2 value	P value
Age (mean, year)	57.50 (8.39)	58.04 (6.26)	− 0.271	0.788
Weight (mean, kg)	68.39 (11.15)	72.21 (10.99)	− 1.292	0.202
Height (mean, cm)	168.50 (6.86)	170.39 (7.71)	− 0.971	0.336
BMI (mean, kg/m2)	23.97 (2.65)	24.73 (2.07)	− 1.191	0.239
Gender			0.080	0.778
male	18	19		
female	10	9		
ASA			0.082	0.960
I	10	11		
II	15	14		
III	3	3		
Hypertension(Y/N)	17/11	15/13	0.292	0.589
Diabetes(Y/N)	19/9	20/8	0.084	0.771
Duration of surgery (mean, min)	122.18 (9.86)	119.93 (9.00)	0.892	0.376
Duration of anesthesia (mean, min)	146.21 (10.28)	142.71 (9.15)	1.346	0.184
Propofol (mean, mg)	912.14 (140.41)	940.71 (201.09)	− 0.616	0.540
Sufentanil (mean, ug)	35.75 (5.8)	37.71 (5.80)	− 1.259	0.214
Remifentanil (mean, ug)	2153.57 (337.96)	2176.79 (378.08)	− 0.242	0.810
Blood loss (mean, ml)	158.21 (20.87)	152.50 (19.56)	1.060	0.294
Infusion volume (mean, ml)	2025.00 (325.91)	2060.36 (367.04)	− 0.381	0.705
Urine volume (mean, ml)	548.57 (90.21)	532.86 (99.21)	0.620	0.538
PACU stay (mean, min)	23.14 (2.14)	22.96 (2.60)	0.281	0.780

**Figure 2:**
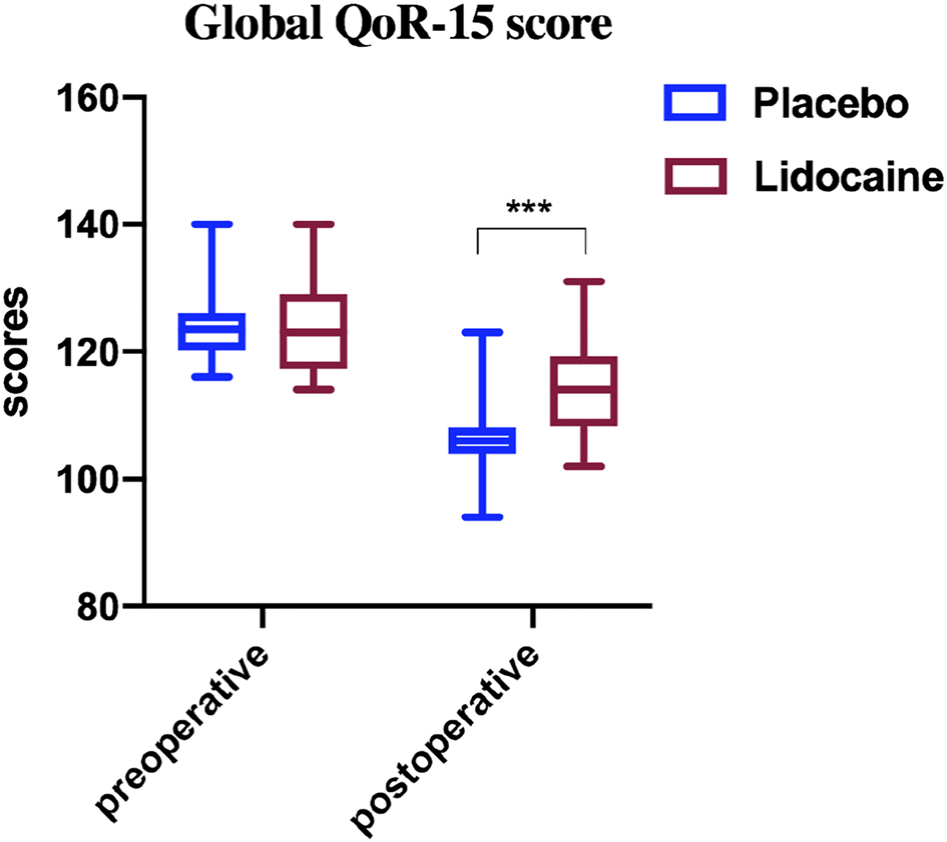
The global QOR-15 scores in patients before or who suffered lumbar spinal stenosis surgery with or without Lidocaine. The scores are presented as median, IQR, and min to max. ***p < 0.001.

As shown in Fig. 2, the global QoR-15 scores were significantly higher 48 hours postoperatively (better recovery) in the lidocaine group than in the placebo group (median 114, IQR 108.25–119.25 compared with median 106, IQR 104–108, *P* < 0.001), the estimated median difference: 8, 95% CI (5 to 11).

**Figure 3:**
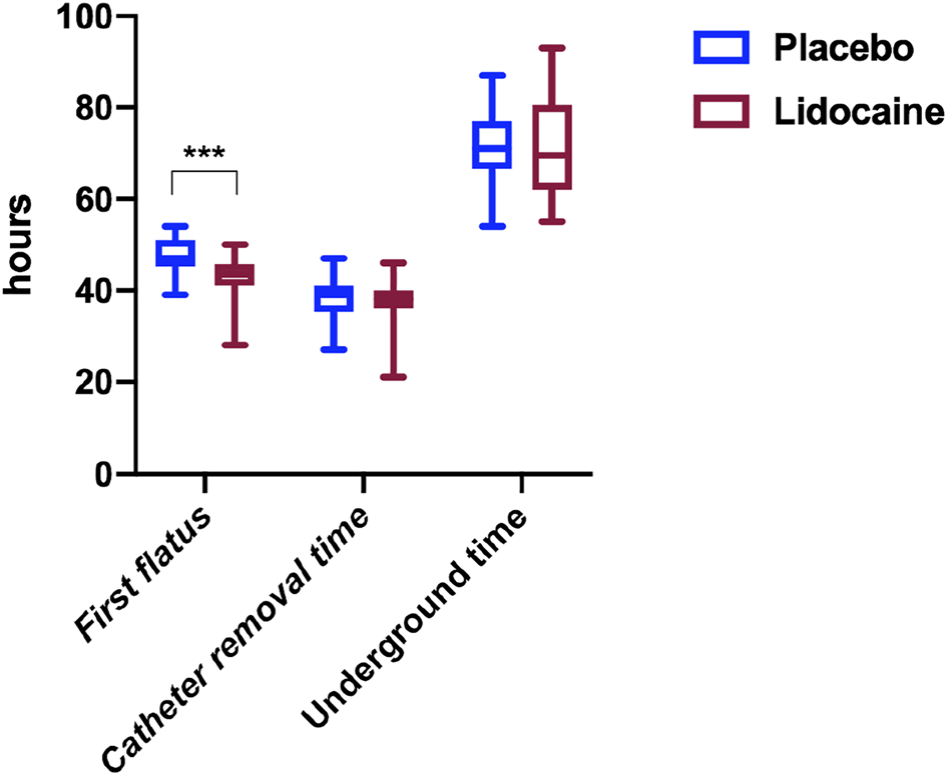
The secondary outcomes of first flatus, catheter removal time, and underground time in patients who suffered lumbar spinal stenosis surgery with or without Lidocaine. The hours are presented as median, IQR, and min to max. ***p < 0.001.

As shown in Fig. 3, the mean time to first flatus in the lidocaine group was significantly shorter than that in the placebo group (median 43.5, IQR 41–45.75, compared with median 47, IQR 45.25–51, *P* < 0.01), the estimated median difference: − 4, 95% CI (− 2 to − 6). The following aspects of the two groups (lidocaine vs placebo) were comparable: catheter removal time (median 38, IQR 36–40, compared with median 39, IQR 35.25–41, *P* = 0.505), the estimated median difference: − 1, 95% (CI − 3 to 2). The underground time (median 69.5, IQR 62–80.5, compared with median 71, IQR 66.5–77, *P* = 0.640), the estimated median difference: − 1, 95% CI (− 7 to 4).

**Figure 4:**
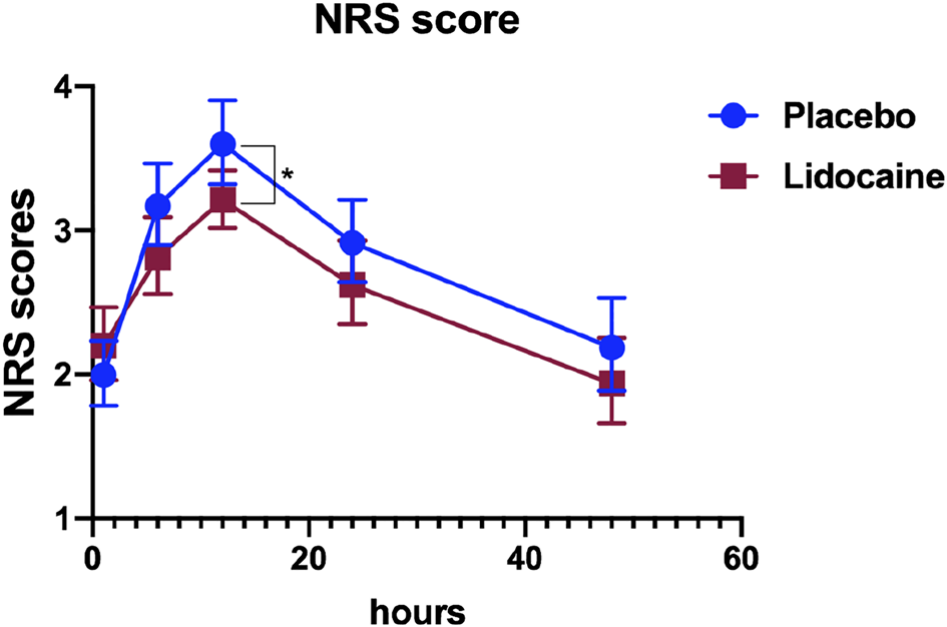
The NRS scores in patients who suffered lumbar spinal stenosis surgery with or without Lidocaine. The scores are presented as mean and standard deviation. *p < 0.05.

Systemic lidocaine could reduce acute NRS pain scores at 12th hours after lumbar spinal stenosis surgery (SD 3.5 (3–4) vs. 3 (3–4), *P* = 0.045). NRS scores at other postoperative time points were not comparable, see Fig. 4.

**Figure 5:**
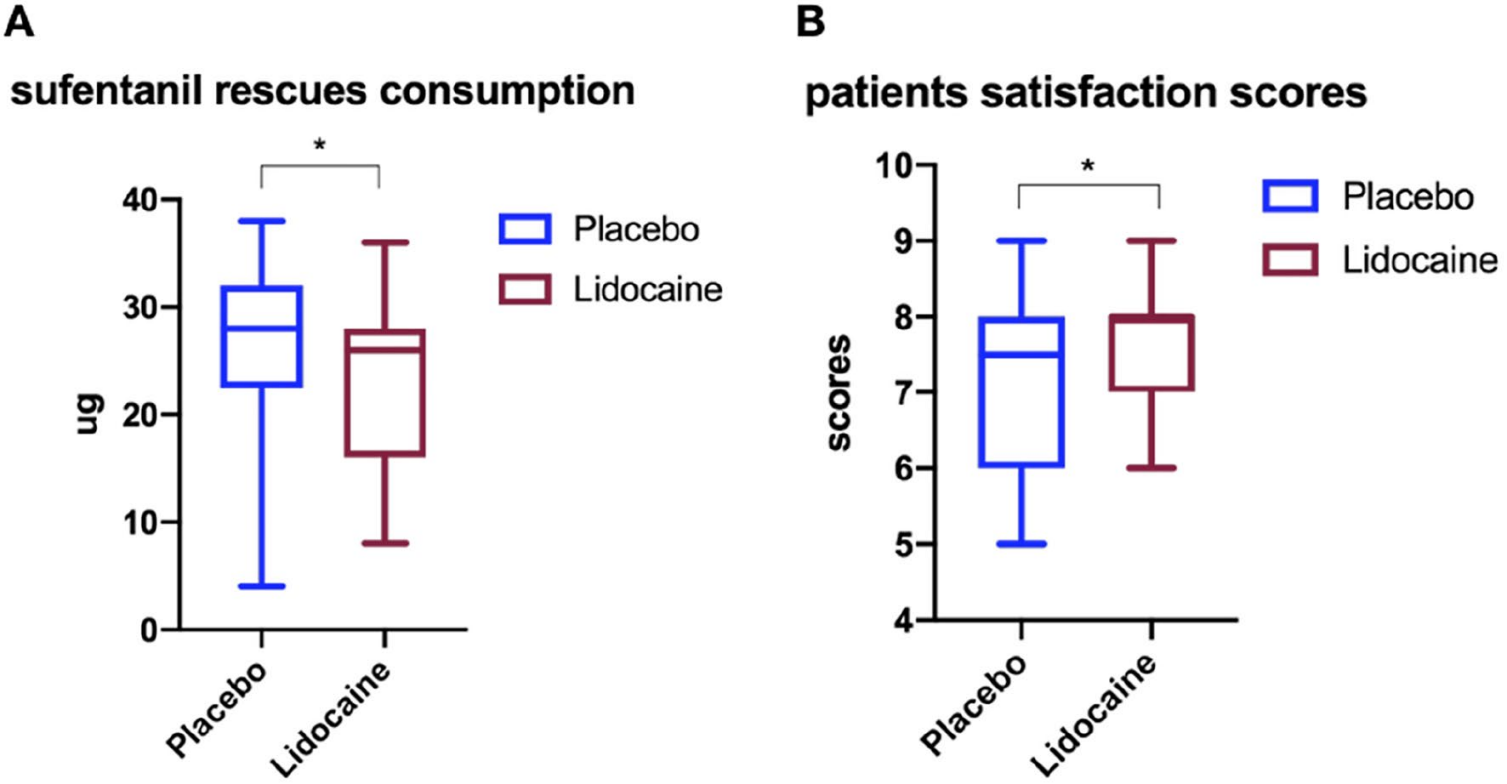
The sufentanil rescues consumption (**A**) and patient satisfaction scores (**B**) when suffering from lumbar spinal stenosis surgery. The dose of sufentanil or scores is presented as median, IQR, and min to max. *p < 0.05.

The placebo group had higher cumulative consumption of opioids (sufentanil) 48 hours after surgery compared with the lidocaine group (median 28 μg, IQR 22.5–32, compared with median 26 μg, IQR 16–28; *P* = 0.046), see Fig. 5A. The Lidocaine group had higher patient satisfaction scores (mean, SD, 7.50 [0.92] vs. 6.93 [1.18]; *P* = 0.049), see Fig. 5B. The median difference between the lidocaine group and the placebo group was 4 μg (95% CI 0 to 8, *P* = 0.046).

**Figure 6:**
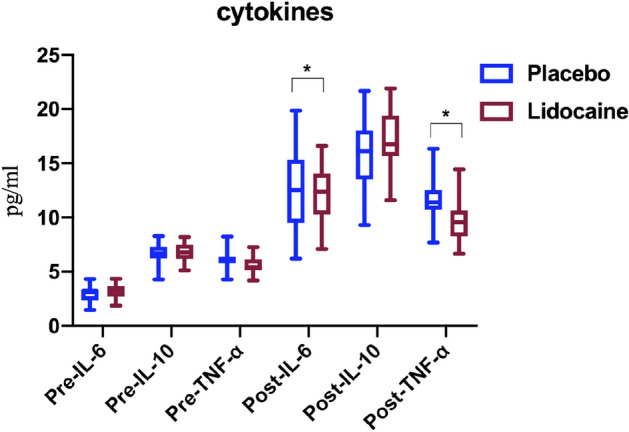
The cytokines in patients before or post-lumbar spinal stenosis surgery with or without Lidocaine. The cytokines are presented as median, IQR, and min to max. Pre-, before the lumbar spinal stenosis surgery. Post-, 48 hours later when patients suffered the lumbar spinal stenosis surgery. *p < 0.05.

As shown in Fig. 6, there was no significant difference in the basal levels of IL-6, IL-10, and TNF-α among the two groups (*P* > 0.05), but the levels of IL-6, IL-10, and TNF-α increased after surgery in both groups. The levels of plasma IL-6 and TNF-α were higher in the placebo group than in the lidocaine group 48 hours after surgery (SD, 11.55 [2.08] vs. 10.37 [1.66], *P* = 0.023, 11.06 [1.29] vs. 10.25 [1.56], *P* = 0.039, respectively), meanwhile, the level of the anti-inflammatory cytokine IL-10 expression was similar between the placebo and lidocaine group (SD 13.61 [2.16] vs. 14.67 [2.04], *P* = 0.064).

The incidence of PONV 24 hours and 48 hours after surgery was 8 (28.6%) in the placebo group versus 5 (17.9%) in the lidocaine group, and 7 (25%) in the placebo group versus 5 (17.9%) in the lidocaine group, and the difference was not statistically significant (*P* = 0.342, *P* = 0.313, respectively), as shown in Table 2. The study-related complications, headache, dizziness, and shivering were not comparable between the two groups. The tinnitus, convulsions, arrhythmia, NID, and NB did not occur in all patients.

**Table 2:** Complications for patients treated with placebo or lidocaine.

	Placebo (n = 28)	Lidocaine (n = 28)	χ2 value	*P* value
Occurrence of PONV (%)				
0-24h	8 (28.6)	5 (17.9)	0.902	0.342
24-48h	7 (25.0)	5 (17.9)	1.018	0.313
Headache (%)	3 (10.7)	2 (7.1)	0.220	0.639
Dizziness (%)	2 (7.1)	3 (10.7)	0.220	0.639
Shivering (%)	6 (21.4)	5 (17.9)	0.113	0.737

PONV, postoperative nausea or vomiting.

## Discussion

In this double-blind, placebo, and randomized controlled trial, we demonstrated that perioperative systemic administration of lidocaine for a loading dose of 1.5 mg/kg for 10 min and then infused at 2.0 mg/kg/hour, creatively continued infusion 48 hours by PCIA with the dose of 40 mg/hour could significantly enhance the post-surgical quality of recovery in patients undergoing lumbar spinal stenosis surgery, by improving the recovery of gastrointestinal function, reducing opioid consumption and decreasing levels of cytokines. Many factors may affect the early postoperative recovery, the early postoperative period is the main concern of this study, including the demographic baseline level of the patient, the time of operation and anesthesia, the dosage of anesthetic drugs, and the retention time of intraoperative blood transfusion, relevant data were collected in the anesthetic recovery room. At the same time, early postoperative pain and gastrointestinal function have also attracted our attention, and inflammatory factors that may affect early recovery have also been detected. These are important factors affecting early postoperative rehabilitation. To the best of our knowledge, this is a forefront randomized controlled study evaluating the effects of perioperative intravenous lidocaine infusion on the early postoperative quality of recovery in LSS patients undergoing lumbar spinal stenosis surgery.

Lumbar spinal stenosis refers to the abnormal structure of the spinal canal caused by primary or secondary factors, narrowing in the spinal canal, and lumbago and leg pain mainly characterized by intermittent claudication^22^. Various forms of the spinal canal, neural tube, and foraminal stenosis, changes in spinal canal volume caused by soft tissue, and stenosis of the dural sac itself cause a series of lumbago and leg pain and a series of neurological symptoms^23^. Other spinal diseases, such as herniated or herniated discs, bone spurs, or spondylolisthesis, can also narrow the space between the spinal canal and the affected part of the spine, resulting in spinal stenosis^24^. Lumbar spinal stenosis surgery and lumbar decompression are some of the main management measures. Postoperative severe pain and dysfunction are the main factors affecting perioperative recovery^25^. Prior nerve damage is possible to occur after lumbar canal decompression, therefore peristalsis may be disrupted resulting in constipation, and functional obstruction. We must be knowledgeable regarding that some complications may present in the early postoperative period following spinal procedures, thereinto brought gastrointestinal dysfunction and bladder urination dysfunction to the forefront^26–28^. Postoperative intestinal dysfunction seriously affects the recovery and quality of life of patients after lumbar spinal stenosis surgery.

Postoperative recovery begins at the time of surgery, only when patients have returned to their baseline function or population standard, it is completed^29^. Perioperative recovery is a complex process that involves many areas, such as symptoms, physical, emotional, social, and economic functioning, and perceived health^30^, especially in the early stages after surgery. However, studies examining interventions often focus on morbidity, postoperative organ dysfunction, or surgical complications, rarely based on postoperative recovery as the primary outcome^31^. QoR-15 was developed from the QoR-40, which was one of the most comprehensive assessment scales to evaluate a patient’s overall health status postoperatively. It has been widely used and validated as a measure of the quality of postoperative recovery, the higher the score, the better the post-surgical recovery^21^. The QoR-15 has the same measurement effect as QoR-40 but is more feasible to use in clinical practice^32^. Previous studies demonstrated that a change of 8.0 points or more on the QoR-15 score was considered a clinically relevant improvement or deterioration^19,20^. The number of patients to be included was calculated on the basis of these studies to reach the conclusion of whether the intervention is effective can be verified while reducing the difficulty of the work as much as possible.

In the present study, the total QoR-15 scores in both groups decreased significantly from baseline, suggesting that surgery and anesthesia significantly negatively impacted postoperative quality. The difference between the lidocaine and placebo groups was 8.0 points. In patients receiving perioperative systemic lidocaine infusion, less moderate-to-severe pain, low NRS scores in the 12^th^ hour, and short median sufentanil rescues consumption request was achieved compared to the placebo group, indicating intravenous lidocaine could reduce pain intensity postoperatively and improve the pain dimension score. Meanwhile, the physical comfort scale of the QoR-15 mainly contains items related to post-surgical adverse effects including physical comfort, physical independence, psychological support, and emotional state^33^. Furthermore, our study found the patients who underwent lumbar spinal stenosis surgery and received systemic lidocaine had a short first flatus time, although the underground time and urine tube pulling time were similar, indicating that lidocaine could shorten the duration of postoperative ileus and accelerate postoperative recovery of intestinal function. It is noteworthy that the postoperative psychological state and satisfaction of patients are related to postoperative recovery. The patients’ satisfaction scores in the lidocaine group were higher compared to the placebo group, and patients felt more comfortable, indicating that systemic lidocaine administration significantly improved postoperative quality of life both physically and psychologically. All of them were the underlying cause of why perioperative systemic lidocaine administration improved patients' postoperative QoR-15 scores.

Muscularis externa inflammation and an influx of leukocytes are associated with the inhibition of postoperative gastrointestinal transport^34^. Prevention of inflammation-related damage to the intestinal wall is currently considered a promising therapeutic strategy to shorten postoperative dyskinesia^35^. Inflammation resolution is a coordinated process that stops neutrophil recruitment, clears damaged areas, and induces collagen deposition, angiogenesis, and recovery of tissue function through the secretion of anti-inflammatory cytokines and lipid mediators^36^. Lidocaine is an adjunctive analgesic, widely used in nerve block and intravenous infusion due to its analgesic, anti-arrhythmic, anti-inflammatory effects, or anti-cancer mediated by a number of molecular mechanisms^37–40^. Intravenous lidocaine has the added advantage of alleviating surgical inflammation, and immune alterations and sparing opioids^21,41^. Systemic administration of lidocaine could alleviate streptozotocin-induced allodynia, supposedly by modulating the p38 pathway in spinal microglia^42^. Subsequently, an in vitro study showed that lidocaine directly acts on microglia by inhibiting the increase of intracellular Ca^2+^ triggered by ATP and p38 MAPK activation. Hence, the production of the pro-inflammatory cytokines, TNF-α, IL-1β, and IL-6, was decreased^43^. In the spinal nerve ligation model of neuropathic pain in rats, systemic lidocaine decreased tactile allodynia, possibly mediated by decreasing pro-inflammatory cytokines^44^.

In addition, reduces complications and accelerates early recovery of postoperative intestinal function, thereby improving the quality of recovery and shortening hospital stays^45^. Therefore, this study shows that lidocaine can relieve inflammation, promote the recovery of intestinal function, and accelerate the overall postoperative quality of recovery in patients undergoing lumbar spinal stenosis surgery. However, the efficacy of lidocaine on the recovery of postoperative ileus has not been studied conclusively. Postoperative ileus is a complex disease with major intrinsic factors including surgical stress (i.e. intestinal processing), secretion of inflammatory mediators and endogenous opioids in the gastrointestinal tract, changes in hormone levels, and electrolyte and fluid balance^46^. Meta-analysis and systematic review show that systemic lidocaine could short the meantime to first bowel movement 7.92 hours than placebo^47^, improve sedation^48^, reduce intraoperative opioids consumption in patients undergoing an elective surgical procedure under general anesthesia^49^, and an animal study indicated lidocaine treatment regimen was an apparent resolution of clinical signs of ileus^50^. However, Yao et al.^51^ do not support using perioperative systemic lidocaine as a potential strategy to improve postoperative pain and enhance QoR-15 in patients undergoing video-assisted thoracic surgery. Preoperative acetaminophen and gabapentin-based analgesic combined with an intraoperative infusion of lidocaine do not improve recovery in patients undergoing multisegmental spine surgery^16^. We believe that the pumping rate and time of lidocaine may be factors affecting postoperative recovery and that focusing on different times after surgery may also lead to different outcomes. In fact, a short time for intraoperative lidocaine infusion can also improve the patient’s recovery score, although this score between the two groups was not yet statistically significant.

The mechanism by which lidocaine accelerates recovery of intestinal function remains unclear. Previous studies have shown that lidocaine could inhibit inflammatory responses which were confirmed by our study, protects epithelial intestinal cells^52^, contract annular and longitudinal smooth muscles^53^, decrease the permeability of lipopolysaccharide jejunum after ischemia, and accelerate the recovery of the mucosal barrier^54^. The other important finding of this study was that intravenous lidocaine inhibited the release of cytokines IL-6 and TNF-α, cytokines widely believed to promote inflammation. However, for cytokine IL-10 and PONV, it had less effect, the expression level and occurrence rate were similar in both groups. The possible reason is that we included a limited number of patients and found no remarkable difference in effects in these enrolled patients.

In addition, as opioids are a mainstay of pain relief and opioid receptors are densely distributed in the gastrointestinal tract, symptoms and side effects are expected in these patients. Postoperatively, particularly with abdominal surgery, opioid-induced ileus may ensue and opioid-induced constipation is common^55^. Opioids inhibit gastrointestinal motility and increase intestinal mucosal permeability, aggravating postoperative ileus^56^. Thus, reducing perioperative opioid use is a real challenge, and one of the goals of enhanced recovery after lumbar spinal stenosis surgery is to reduce potential opioid-related side effects. Similar to previous studies, the present study showed a reduction in the total amount of sufentanil used for postoperative rescue in patients receiving lidocaine. This may be a potential mechanism to enhance patients' postoperative QoR-15 score in patients undergoing lumbar spinal stenosis surgery.

Neuropathic bowel dysfunction and neuropathic bladder, which are major consequences of spinal cord injury and occasionally degenerative lumbar disease, were not observed in our study in both groups^28^. Actually, intravenous lidocaine can cause a variety of adverse reactions, including numbness of the tongue and lips, tinnitus, dizziness, convulsions, and arrhythmias^57^. In our study, although few patients had bradycardia or tachycardia during the surgery, the electrocardiogram showed normal sinus rhythm, and last for a short time. Therefore, we did not classify lidocaine-related side effects. None of the patients experienced convulsions, persistent arrhythmias, or other serious adverse events during the procedure. No side effects related to lidocaine were observed during the postoperative follow-up period.

### Limitations

First, we only assessed the effects of systemic lidocaine on postoperative quality of recovery on the early recovery for 48 hours, studies need to focus on longer observation periods for long-term quality of recovery in the future. Second, we did not directly measure the concentrations of lidocaine in patients during the procedure and postoperative follow-up period and performed our study based on doses of lidocaine used in previous studies in the literature. Third, this study did not explore the effect of lidocaine infusion speed and dose on lumbar spinal stenosis surgery, the optimal dosage and speed of lidocaine to accelerate postoperative quality recovery of lumbar spinal stenosis surgery remains to be further studied. Fourth, the study did not distinguish between specific surgical procedures for lumbar spinal stenosis, and only selected open surgery with a posterior approach. Although different surgeries are similar in basic operation, it is true that different surgical methods may have a certain impact on the quality of postoperative recovery. Last but not least, the present study was a single-center clinical trial, whereas the clinical promotion of systemic lidocaine in lumbar spinal stenosis surgery needs a multi-center study.

## Conclusions

Under the conditions of the present study, perioperative systemic lidocaine enhanced the postoperative quality of recovery for patients undergoing lumbar spinal stenosis surgery, indicating that it may be a useful intervention to aid recovery following the early stages of the end of lumbar spinal stenosis surgery. Meanwhile, participants in the lidocaine group met early postoperative gastrointestinal function recovery, superior pain relief, fewer cytokines, and postoperative cumulative consumption of opioids, the complication of systemic lidocaine was a no-show.

## Data Availability

The datasets generated and analyzed during the current study are available from the corresponding author upon reasonable request.
