# Author Correction: Effect of systemic lidocaine on postoperative quality of recovery, the gastrointestinal function, inflammatory cytokines of lumbar spinal stenosis surgery: a randomized trial

**DOI:** 10.1038/s41598-024-59219-9

**Published:** 2024-04-11

**Authors:** Yu Wu, Zhuoming Chen, Caimiao Yao, Houxin Sun, Hongxia Li, Xuyang Du, Jianzheng Cheng, Xiaojian Wan

**Affiliations:** 1 Department of Anesthesiology, Bethune International Peace Hospital, Shijiazhuang, 050082 China; 2 School of Textile and Fashion, Shanghai University of Engineering Science, Shanghai, 201620 China; 3 Department of Clinical Laboratory, Bethune International Peace Hospital, Shijiazhuang, 050082 China; 4 Department of Anesthesiology and Critical Care Medicine, Changhai Hospital, Naval Medical University, Shanghai, 200433 China

Correction to: *Scientific Reports* 10.1038/s41598-023-45022-5, published online 17 October 2023

The Funding section in the original version of this Article was incomplete.

“The Science and Technology Research Program of Hebei Provincial Health Commission (20231335); The project of Hebei Provincial Administration of Traditional Chinese Medicine (202339).”

now reads:

“The Science and Technology Research Program of Hebei Provincial Health Commission (20231335); The project of Hebei Provincial Administration of Traditional Chinese Medicine (202339). The Incubator Program for Startups of the 980th Hospital of the Joint Logistic Support Force (FYJHMS-04)."

The original Article has been corrected.

